# Two Inducible Prophages of an Antarctic *Pseudomonas* sp. ANT_H14 Use the Same Capsid for Packaging Their Genomes – Characterization of a Novel Phage Helper-Satellite System

**DOI:** 10.1371/journal.pone.0158889

**Published:** 2016-07-07

**Authors:** Lukasz Dziewit, Monika Radlinska

**Affiliations:** 1 Department of Bacterial Genetics, Institute of Microbiology, Faculty of Biology, University of Warsaw, Warsaw, Poland; 2 Department of Virology, Institute of Microbiology, Faculty of Biology, University of Warsaw, Warsaw, Poland; Centro Nacional de Biotecnologia—CSIC / CIF Q2818002D, SPAIN

## Abstract

Two novel prophages ФAH14a and ФAH14b of a psychrotolerant Antarctic bacterium *Pseudomonas* sp. ANT_H14 have been characterized. They were simultaneously induced with mitomycin C and packed into capsids of the same size and protein composition. The genome sequences of ФAH14a and ФAH14b have been determined. ФAH14b, the phage with a smaller genome (16,812 bp) seems to parasitize ФAH14a (55,060 bp) and utilizes its capsids, as only the latter encodes a complete set of structural proteins. Both viruses probably constitute a phage helper-satellite system, analogous to the P2-P4 duo. This study describes the architecture and function of the ФAH14a and ФAH14b genomes. Moreover, a functional analysis of a ФAH14a-encoded lytic enzyme and a DNA methyltransferase was performed. *In silico* analysis revealed the presence of the homologs of ФAH14a and ФAH14b in other *Pseudomonas* genomes, which may suggest that helper-satellite systems related to the one described in this work are common in pseudomonads.

## Introduction

Bacteriophages not only outnumber all other viruses, but they are also the most abundant, diverse and widely distributed biological entities in the biosphere. They are a valuable source of enzymes that serve as important tools in molecular genetics and biotechnology [1]. After infecting the host cell, temperate phages can choose between a lytic and lysogenic pathway of development. In the lysogenic cycle, a virus often integrates its genome into the chromosome of the host cell and, as a prophage, remains dormant until induction [2].

Prophages and prophage remnants have been identified in many bacterial genomes sequenced so far, suggesting that this group of mobile genetic elements is widespread in bacteria and constitutes the main source of genetic diversity and strain variation [3]. Prophages genes can modulate fitness and lifestyle, including virulence, antibiotic tolerance and biofilm formation of their bacterial hosts. Prophage segments can also confer immunity or exclusion, protecting the carrier strain against superinfection [4]. Apart from the fully functional prophages that can be induced to lytic growth, additional types of prophage-related entities have been characterized, i.e. defective and satellite prophages, bacteriocins and gene transfer agents [3].

Satellite phages carry autonomous replication modules, which lack the morphogenesis and structural virion-encoding genes, but they are otherwise functional phages. They use the structural proteins supplied by another ‘helper’ virus for assembly of their own virions, and thus, for their propagation and spread. The best studied examples of such parasitic relationships are those between the Enterobacteria satellite phage P4 (or the related retronphage φR73) and the fully functional phage P2 [5, 6], and *Staphylococcus aureus* phages, where genetic elements called pathogenicity islands (SaPIs) are mobilized by specific helper phages and are packaged into phage-like transducing particles using hijacked structural proteins of the helper phage [7, 8]. In both cases, the expression of the satellite phage genes is strictly regulated to take advantage of the lytic cycle of the helper phage and to maximize the transduction of progeny. This biological phenomenon is often referred to as molecular piracy [9].

The existence of satellite phages seems to be quite common. Many *S*. *aureus* genomes contain one or more SaPIs, and they are probably widespread among other Gram-positive bacteria [7, 8]. Moreover, BLAST searches revealed P4-like elements in the genomes of a number of Enterobacteria, including members of the genera *Escherichia* [10], *Shigella* [11] and *Salmonella* [12]. However, similar systems exploiting helper phages have not been described yet.

The members of the genus *Pseudomonas* demonstrate a great deal of metabolic diversity, and consequently are able to colonize a wide range of niches [13]. Pseudomonads are commonly found in soil, ground water, plants and animals. Currently, GenBank contains 1850 complete genomes of different phages, approximately 7% of which are found within the representatives of the *Pseudomonas* genus. Moreover, numerous prophage sequences have been identified within various *Pseudomonas* spp. genomes [14]. Most of the described bacteriophages that infect pseudomonads are members of the order *Caudovirales*, i.e., they have a head-and-tail morphology and contain double-stranded DNA [15, 16].

Although to date numerous species of the genus *Pseudomonas* have been found in Antarctic water and soil samples, to our knowledge, no cold-active *Pseudomonas* phages originating from that area have so far been described. The lack of known representatives of temperate phages of polar *Pseudomonas* spp. encouraged us to screen the bacterial isolates derived from Antarctic soil using the chemical induction approach.

In this study, we report the isolation and characterization of two mitomycin-inducible prophages of Antarctic *Pseudomonas* sp. ANT_H14, designated ФAH14a and ФAH14b, that use the same viral capsid build of structural proteins encoded by the former one. We propose that they constitute an example of a new bacteriophage satellite-helper system. According to the novel, universal approach of bacteriophage naming [17], the suggested names of ФAH14a and ФAH14b shall be vB_Psp_AH14a and vB_Psp_AH14b, respectively.

## Materials and Methods

### Bacterial strains, plasmids, media, and growth conditions

*Pseudomonas* sp. ANT_H14 was isolated from a soil sample collected near the Arctowski Polish Antarctic Station located on King George Island, the largest of the South Shetland Islands, situated 120 km off the coast of Antarctica. The strain was identified based on the 16S rRNA gene sequencing using universal primers 27f and 1492r [18]. No permission was required for soil samples collection, as the Arctowski Polish Antarctic Station is located outside the Antarctic Specially Protected Area. Moreover, the field study did not involve endangered or protected species.

Other strains used in this study were: *Escherichia coli* TOP10 (Thermo Fisher Scientific, Waltham, MA, USA) and ER2566 (New England BioLabs, Ipswich, MA, USA), *Pseudomonas aeruginosa* PAO1 [19] and seven *Pseudomonas* spp. strains isolated from the same environment as *Pseudomonas* sp. ANT_H14. The stains were cultured under standard conditions in LB medium at 37°C (*E*. *coli* and *P*. *aeruginosa* PAO1) or 22°C (*Pseudomonas* environmental isolates).

When required, growth media were supplemented with kanamycin (Km, 50 μg ml^−1^) and glucose (1%). The vector pET30a (Km^r^, Novagen, Inc., Madison, WI, USA) was used in recombinant protein expression experiments.

### Standard molecular biology procedures

Standard DNA manipulations were carried out according to the protocols described by Sambrook and Russell [20]. Total DNA was isolated from *Pseudomonas* sp. ANT_H14 using a genomic DNA purification kit (Thermo Fisher Scientific, Waltham, MA, USA).

PCR reactions were performed with Phusion High Fidelity DNA polymerase (Thermo Fisher Scientific, Waltham, MA, USA). The amplified DNA fragments were analyzed by agarose gel electrophoresis and, if necessary, purified using a Gel Out kit (Thermo Fisher Scientific, Waltham, MA, USA). Subsequently, the PCR products were digested with restriction enzymes and cloned into appropriate vectors. All the constructs were confirmed by DNA sequencing. Restriction digest assay was performed in a 20-μl reaction volume under conditions recommended by the manufacturer using 0.3 μg of the phage DNA and 10 U of a restriction endonuclease (REase). The test for the presence of cohesive ends of the phage genome was performed as previously described [21], using the following REases: HindIII, SalI, EcoRI, Eco32I and PstI (Thermo Fisher Scientific, Waltham, MA, USA).

### Induction, purification of phage particles, and phage DNA preparation

*Pseudomonas* phages were induced using mitomycin C (Sigma-Aldrich, St. Louis, MO, USA). The bacterial culture was grown to an optical density of 0.4 at 600 nm (OD_600_). The culture was then treated with mitomycin C (500 ng ml^−1^), and its growth (with shaking) was continued for 20 h. Growth and lysis of the bacterial cultures was monitored by hourly measurements of OD_600_. As cell lysis was not observed, it was induced by the addition of chloroform (1 %, v/v). Phage particles were purified from the lysate by PEG/NaCl precipitation [20]. After centrifugation (25,000 × g, 10 min, 4°C), the sediment was suspended in SM buffer (100 mM NaCl, 10 mM MgSO_4_, 50 mM Tris-HCl, pH 7.5). The remaining suspension of the phages was mixed with CsCl (final concentration of 0.7 g ml^-1^) and centrifuged at 150,000 × g for 24 h at 4°C using a Beckman 50.2 Ti rotor (Beckman Coulter, Fullerton, CA).

The visible viral band was collected, diluted 1:10 in SM buffer, and centrifuged in the Beckman 50.2 Ti rotor for 2 h at 110,000 × g at 4°C. The pelleted bacteriophage particles were resuspended in SM buffer. Phage DNA was isolated by treatment with 50 μg ml^-1^ proteinase K and SDS (a final concentration 0.5%) and incubated for 1 h at 56°C, followed by phenol-chloroform extraction and isopropanol precipitation [20]. The obtained DNA was then analyzed by 0.7% agarose gel electrophoresis.

### Electron microscopy

Phage particles were negatively stained with 2% uranyl acetate and electron micrographs were captured with a LEO 912AB transmission electron microscope (Zeiss, Jena, Germany) at 80 kV with a magnification of 100,000×. Triplicate grids were prepared. One hundred viruses per grid were analyzed.

### Tests for lytic growth

To determine bacterial susceptibility to phage-mediated lysis, *Pseudomonas* strains—*P*. *aeruginosa* PAO1 and seven *Pseudomonas* spp. isolates from the Antarctic soil [all the analyzed strains were negative for the presence of ФAH14a and ФAH14b prophages, what was confirmed by PCR analysis with prophage specific oligonucleotide primers (5’CAAGCAGGCCAACATTTACTGCTG3’ and 5’GATCGCTTTGATCGGATAACGCTTGG3’) and (5’GCATGAACGCTATCGTCCTGATCC3’ and 5’GTTCATCGCCGATCATGAGCATAGC-3’)] were grown in liquid LB medium and plated onto LB agar plates. After drying, a drop of the phage suspension was placed on the bacterial layer and incubated at 22°C or 37°C (*P*. *aeruginosa* PAO1 only). The plates were examined for the presence of clear zones indicating bacterial lysis for 18–72 h.

### Phage structural protein analysis

Phage structural proteins were analyzed by SDS-PAGE as previously described [21]. After electrophoresis, the protein bands were visualized by staining the gel with Coomassie brilliant blue R-250 dye and identified by liquid chromatography coupled with mass spectrometry (LC–MS/MS) at the Mass Spectrometry Laboratory, Institute of Biochemistry and Biophysics, Polish Academy of Sciences (IBB PAS, Warsaw, Poland).

### Cloning, overexpression, purification, and testing of the activity of a putative DNA methyltransferase

DNA encoding a putative methyltransferase gene was amplified by PCR using primers (5’-GTTGTTCATATGAAACAGCATCGCGTTTTG-3’ and 5’- GTTGTTGTCGACGGCTGCTGGGTGTTGCACTG-3’) that appended the NdeI and SalI sites (underlined) at the 5′ and 3′ ends of *AH14a_p05*, respectively. The amplified fragment was cleaved with NdeI and SalI and cloned into the NdeI/XhoI-digested pET30a, yielding pET-AH14a_p05. The recombinant enzyme was expressed in the *E*. *coli* strain ER2566. Protein expression and restriction enzyme digestion protection assay were performed as previously described [22].

### Cloning, overexpression, and testing the activity of a putative hydrolase

DNA encoding a putative hydrolase gene was amplified by PCR using primers (5’-GAAGAACATATGACTGAATCCGAAAAAGAC-3’ and 5’-GAAGAACTCGAG CGGCACATCCTTGAAGAAC-3’, the appended NdeI and XhoI sites were underlined) at the 5′ and 3′ ends of *AH14a_p93*, respectively. The obtained DNA fragment was cleaved with NdeI and XhoI and cloned into the NdeI/XhoI-digested pET30a, yielding pET-AH14a_p93. The plasmid pET-AH14a_p93 was introduced into *E*. coli ER2566, and the resulting strain was cultured to optical density of 0.4 at OD_600_ in LB medium supplemented with Km and glucose (to repress the basal expression from the T7 promoter). At that point, the culture was centrifuged, resuspended in fresh LB medium, and divided into two equal parts, of which one was supplemented with glucose and the other with isopropyl-β-d-thiogalactopyranoside (IPTG) to a final concentration of 1 mM. Growth of these two cultures was monitored by measuring the optical density. In a parallel control test, the plasmid construct pET-AH14a_p05 was used.

### DNA sequencing

The complete nucleotide sequences of ΦAH14a and ΦAH14b were determined at the Laboratory of DNA Sequencing and Oligonucleotide Synthesis, IBB PAS (Poland). The phage genomes were sequenced on the Illumina MiSeq instrument in paired-end mode using v3 chemistry kit. The genome of each virus was obtained as a single contig with 3390 reads and 17.2 coverage for ΦAH14a and 5160 reads and 83.8 coverage for ΦAH14b. The obtained sequence reads were filtered for quality and assembled using Newbler v3.0 software (Roche). The end closing was performed by PCR and subsequent sequencing of the PCR products.

### Bioinformatics

Bioinformatic characterization of the nucleotide sequence of ΦAH14a and ΦAH14b was performed using Clone Manager 8 (Sci-Ed) and Artemis software [23]. The genomes were automatically annotated using the RAST server [24] and the resulting annotations were then thoroughly manually curated. BLASTP [25] and Psi-BLAST algorithms were used for the similarity searches in the National Center for Biotechnology Information (NCBI) database (http://www.ncbi.nlm.nih.gov). Moreover, similarity searches were performed using the UniProt (http://www.uniprot.org/), Pfam (http://pfam.xfam.org/) HHpred [26] and REBASE databases [27]. Putative tRNA genes were identified using the tRNAScan-SE [28] and ARAGORN programs [29]. A stand-alone version of BLASTP (2.2.30+) was used to examine the similarity of amino acid sequences encoded by ФAH14a and ФAH14b and other bacteriophages. Protein motifs were scanned at the Prosite server http://www.expasy.org/prosite [30]. Prophage sequences within the genomes were identified using PHAge Search Tool (PHAST) [31] and by manual inspection. A phage family search was carried out using VIRFAM [32].

### Nucleotide sequences accession numbers

The nucleotide sequences of ΦAH14a and ΦAH14b, determined in this study, have been annotated and deposited in the GenBank database with the accession numbers KU708004 and KU708005, respectively.

## Results and Discussion

### Morphology of the viral particles

**Fig 1:**
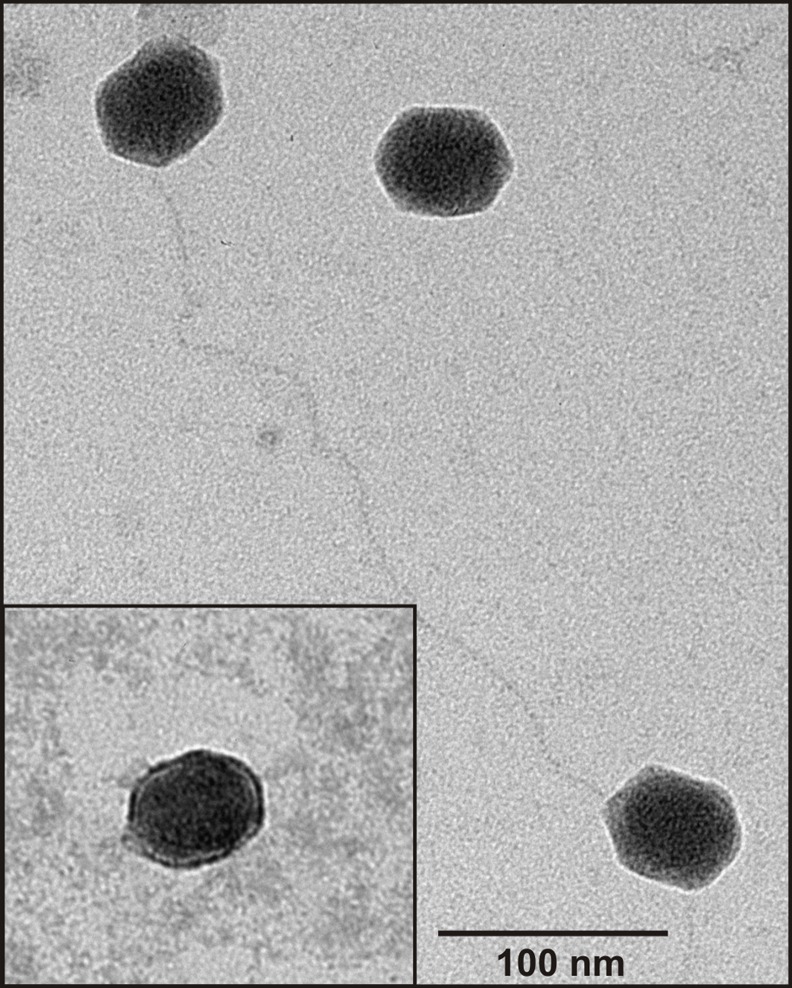
Electron micrograph of the viral particle mixture obtained from a mitomycin C-induced culture of the strain *Pseudomonas* sp. ANT_H14. Samples were stained with 2% uranyl acetate. The scale bar represents 100 nm.

An exponentially growing culture of *Pseudomonas* sp. ANT_H14 was exposed to the prophage-inducing chemical mitomycin C. The resulting lysate was purified by PEG precipitation and CsCl density gradient separation. The visible band was collected and analyzed for the presence of phage particles by transmission electron microscopy (TEM). Electron micrographs consistently showed that viral particles were of uniform size and had slightly elongated heads (about 60nm length and 55-nm width). No attached tails and fibers were observed (Fig 1).

DNA was extracted from bacteriophage particles and subjected to high throughput sequencing. The resulting reads were successfully assembled into two separate contigs comprising 55,060 bp and 16,812 bp, respectively.

**Fig 2:**
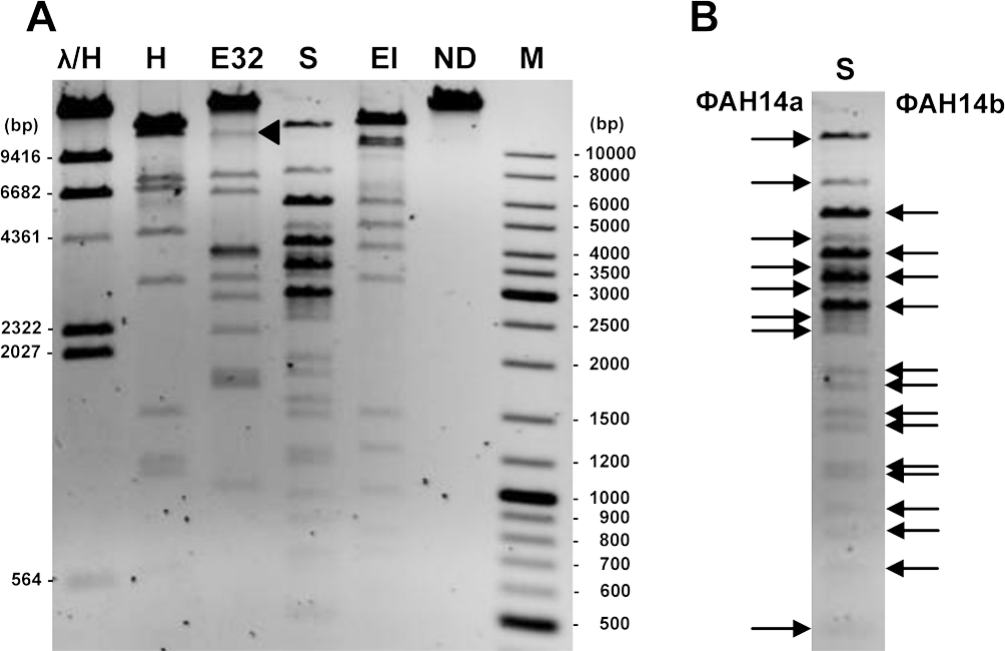
Restriction patterns of DNA extracted from purified virions cleaved with the selected REases. Panel A: HindIII (H), Eco32I (E32), SalI (S) and EcoRI (EI). ND, undigested DNA. M, GeneRuler 100- to 10,000-bp size marker. λ/H, λ DNA cleaved with HindIII. ~14 kb restriction fragment of EcoR32I digested virion DNA is marked with a triangle. Panel B: Arrows indicate restriction fragments assigned to ФAH14a (left) and ФAH14b (right) genomic DNA, obtained by SalI digestion.

In the next step, phage DNA was treated with various restriction enzymes and the resulting band patterns obtained after gel electrophoresis were compared with the predicted digestion profiles (Fig 2). Comparison of the observed and predicted DNA digestion patterns indicated that each of the visible restriction fragments could be assigned to one of the two contigs. Analysis of these results supports the earlier conclusion on the physical separation of these two molecules. At this stage, we concluded that the *Pseudomonas* sp. ANT_H14 strain harbors two inducible prophages that were named ФAH14a and ФAH14b, respectively.

Moreover, the comparison of the restriction profiles of both phage DNAs with their nucleotide sequences yielded circular restriction maps of the phage genomes, suggesting that the linear DNA molecules of ФAH14a and ФAH14b are circularly permuted.

Heat treatment of the restriction fragments followed by either rapid or slow cooling did not alter the restriction patterns, excluding the possibility of cohesive genome ends (where unit length genomes are cut from products of rolling-circle replication—concatemers and packaged). Therefore, it can be assumed that ФAH14a and ФAH14b DNAs were packaged by a headful mechanism (*pac* type), in which the sequence independent cleavage of the DNA is determined by the amount of DNA packaged. The headful mechanism is characteristic for circularly permuted genomes [33].

Interestingly, the densitometry analysis of the DNA bands assigned to the larger contig (ФAH14a) and those assigned to the smaller contig (ФAH14b) indicates that the molar ratio of these two DNA molecules in the mixture is approximately 1:3 (Fig 2). We hypothesized that the ФAH14a and ФAH14b genomes are not packaged together into the same viral particle and the observed molar ratio of ФAH14a:ФAH14b is probably the result of packaging the ФAH14b DNA at least as trimers into the ФAH14a capsids by a headful mechanism. Three lengths of the ФAH14b genome roughly correspond to the length of the monomeric ФAH14a genome. This interpretation is supported by the restriction analysis: (i) there is only one band in the lane containing undigested DNAs (a ~16.8 kbp monomer of ФAH14b would migrate faster than a three times larger concatamer) [Fig 2 –line ND]; (ii) the uncut ФAH14b DNA molecule that lacks sites for Eco32I REase migrates similarly to the undigested ФAH14a (~55 kb), but much slower than the largest restriction fragment ФAH14a/Eco32I (~14 kb) [Fig 2: comparison of line E32 and line ND]; (iii) ФAH14b cut with EcoRI or HindIII (one site) migrates to the same position as the other restriction fragments with ~ 15 kb size (e.g. ФAH14a/SalI), which corresponds to the size of the monomeric ФAH14b genome [Fig 2: comparison of line EI (or H) and line S].

Identical results of both restriction and densitometry analyses were obtained for five independent induction experiments. This led us to a surprising conclusion that the distribution of ФAH14a monomeric and ФAH14b multimeric (most probably trimeric) DNA molecules in the capsids is equal. As it was previously reported, usually only one phage can be recovered after induction of poly-lysogenic strains or the productivity of at least one phage declines, which is probably a consequence of the competition between the co-infecting phages [34, 35]. Therefore, the further experimental work is needed to elucidate the phenomenon of ФAH14a and ФAH14b equal productivity.

### Tests for lytic growth

*Pseudomonas aeruginosa* PAO1 and seven environmental isolates of *Pseudomonas* spp. from Antarctic soil were tested as potential hosts for ФAH14a and ФAH14b by a spot test. None of the tested bacterial strains supported detectable lytic growth of either of the phages. *Pseudomonas* sp. ANT_H14 strain was immune to infection by ФAH14a and ФAH14b, which was not surprising, as it is a ФAH14a and ФAH14b lysogen.

### General features of the ФAH14a and ФAH14b genomes

The genome of ФAH14a consisted of a linear double-stranded DNA of 55,060 bp with a 58.1% G+C content. The prediction of the function of each gene was carried out by the comparison of the amino acid sequences of their products with known protein sequences using the BLAST program. Based on the *in silico* analysis, 28 out of the 95 identified open reading frames (ORFs) were assigned putative functions, while the remaining 67 ORFs exhibited similarity to uncharacterized proteins.

**Fig 3:**
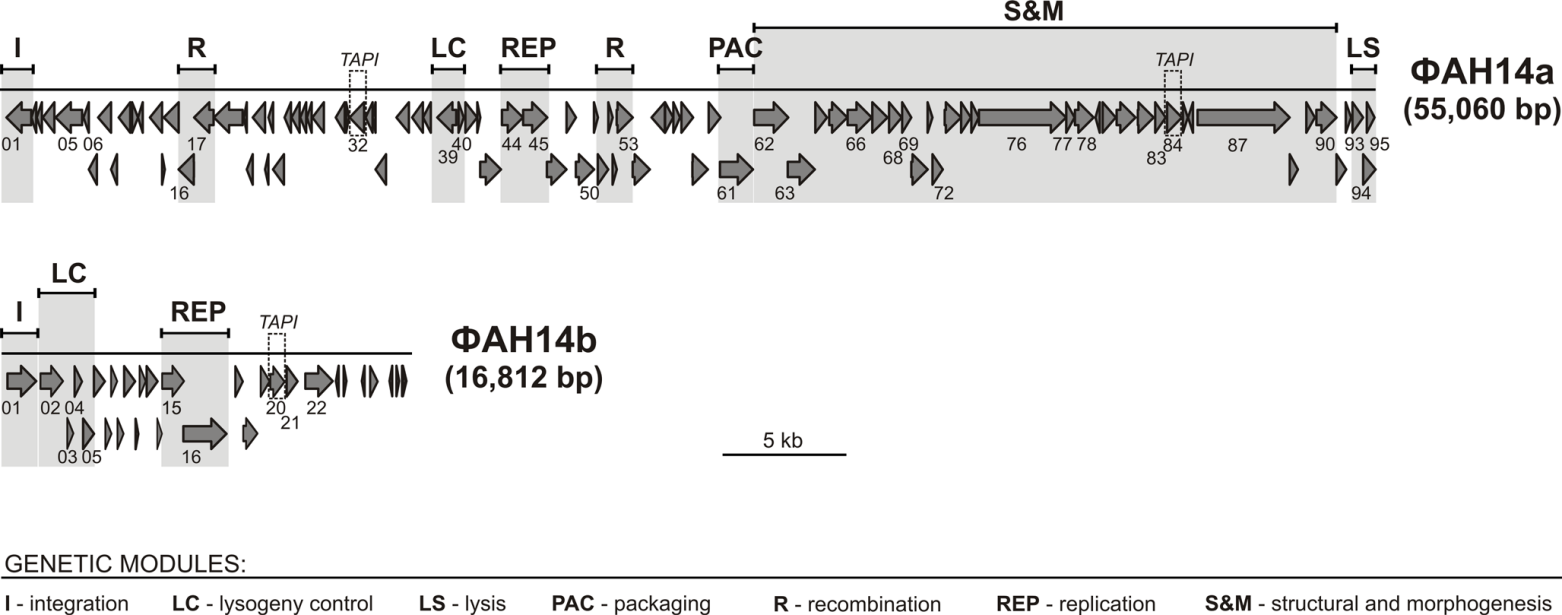
Genome organization of the prophages ΦAH14a and ΦAH14b of *Pseudomonas* sp. ANT_H14. Arrows indicate the transcriptional orientation of the genes. The gray shaded blocks represent genetic modules identified within the prophages. The genes encoding tail assembly protein I (TAPI) are indicated.

Genes located upstream of the lysis-lysogeny module of ФAH14a were found on the lower strand, while those upstream of the position 18,356 were found on the upper strand (Fig 3). No tRNA genes were detected. Positions, sizes and putative functions of the proteins are listed in Table 1.

**Table 1:** Genes located within the ФAH14a genome and proteins homologous to the ФAH14a-encoded proteins found in selected *Pseudomonas* spp. genomes and *Pseudomonas* phages. Searches were performed with the following cut offs *E* value < 1e-10 and < 1e-40. The hits obtained with e-value 1e^-40^ are bolded.

ORF no.	Coding region (bp)	Strand	Protein size (aa)	Possible function	*Pseudomonas* sp. TKP ^a^	*Pseudomonas brassicacearum* subsp. brassicacearum NFM421^b^	*Pseudomonas mosselii* SJ10^c^	*Pseudomonas cichorii* JBC1^d^	*Pseudomonas fluorescens* A506^e^	*Pseudomonas* phage YMC11/02/R656^f^	*Pseudomonas* phage phiPSA1^g^
1	262–1251	←	329	Integrase							
2	1263–1457	←	64	Hypothetical protein							
3	1475–1678	←	67	Hypothetical protein		WP_013693913			WP_014717866		
4	1715–2179	←	154	Hypothetical protein							
5	2229–3293	←	354	C4-methyl-C DNA methyltransferase							**YP_009043573**
6	3343–3561	←	72	Transcriptional regulator							
7	3558–3884	←	108	Hypothetical protein							YP_009043571
8	3931–4449	←	172	Hypothetical protein							
9	4446–4685	←	79	Hypothetical protein							
10	4753–5220	←	155	Hypothetical protein		AEA70234					
11	5293–5526	→	77	Hypothetical protein							
12	5527–5718	←	63	Hypothetical protein	WP_024075235						
13	6003–6491	←	162	Hypothetical protein		WP_013693901					
14	6490–6603	→	37	Hypothetical protein							
15	6561–7154	←	197	Hypothetical protein		**AEA70226**	WP_023629046				
16	7151–7768	←	205	Exonuclease		**WP_013693899**				YP_009187414	
17	7772–8554	←	260	ERF-like, single-stranded DNA-binding protein							
18	8562–9719	←	385	Hypothetical protein						**YP_009187412**	**YP_009043578**
19	9765–9884	←	39	Hypothetical protein							
20	9881–10120	←	79	Hypothetical protein							
21	10117–10614	←	165	Hypothetical protein						YP_009187406	
22	10611–10748	←	45	Hypothetical protein							
23	10745–10936	←	63	Hypothetical protein	WP_024075239	WP_013693895					
24	10933–11370	←	145	Hypothetical protein	**WP_041160924**				WP_014717881		
25	11392–11652	←	86	Hypothetical protein							
26	11672–11998	←	108	Hypothetical protein							
27	12020–12262	←	80	Hypothetical protein							
28	12291–12482	←	63	Hypothetical protein							
29	12511–12969	←	152	Hypothetical protein							
30	13444–13803	←	119	Hypothetical protein							
31	13859–13975	→	38	Hypothetical protein							
32	13998–14579	←	193	Tail assembly protein I	**WP_024075287**	**WP_013693842**	**WP_038706192**	**WP_025261068**	**WP_014717927**	**YP_009187448**	
33	14637–14918	←	93	Hypothetical protein	WP_024075286				WP_014717926		
34	14923–15036	←	37	Hypothetical protein							
35	15036–15452	←	138	Hypothetical protein							
36	15867–16370	←	167	Hypothetical protein							
37	16496–16933	←	145	Transcription regulator							
38	16936–17250	←	104	Hypothetical protein							
39	17503–18264	←	253	CI-like transcription repressor		AEA70212			**WP_014717888**		YP_009043581 YP_009043583
40	18356–18565	→	69	Cro-like repressor protein		WP_013693885					
41	18630–19100	→	156	Hypothetical protein					**WP_014717889**		
42	19105–19227	→	40	Hypothetical protein							
43	19224–20057	→	277	Hypothetical protein							
44	20094–20939	→	281	ParBc-like nuclease							
45	20941–21909	→	322	Replication protein	**WP_024075254**	**WP_013693882**		**WP_025261098**	**WP_014717891**		
46	21896–22681	→	261	Hypothetical protein	**WP_041160925**	**WP_013693881**		**WP_025261097**	**WP_044483149**		
47	22678–23058	→	126	Hypothetical protein							
48	23055–23783	→	242	Hypothetical protein							
49	23780–23935	→	51	Hypothetical protein							
50	23932–24348	→	138	NinB protein			WP_023628606				YP_009043587
51	24348–24527	→	59	Hypothetical protein							
52	24524–24691	→	55	Hypothetical protein							
53	24688–25332	→	214	NinG protein	**WP_024075257**	**WP_013693878**	WP_038707549		WP_014717894	**YP_009187491**	**YP_009043588**
54	25329–26015	→	228	Hypothetical protein		WP_013693876	WP_023628603		WP_014717896		
55	26084–26614	←	176	Hypothetical protein							
56	26628–26951	→	107	Peptidase M48							
57	26953–27225	→	90	Hypothetical protein							
58	27278–27748	→	156	Proteasome subunit beta	WP_024075261		WP_023628598				
59	27714–28337	→	207	Hypothetical protein	**WP_024075263**	**WP_013693867**	**WP_038706194**		**WP_014717904**	**YP_009187479**	**YP_009043592**
60	28369–28845	→	158	Hypothetical protein	**WP_024075264**	**WP_013693866**	**WP_023628595**		**WP_014717905**	**YP_009187478**	
61	28820–30142	→	440	Terminase	**WP_024075265**	**WP_013693865**	**WP_031314408**		**WP_014717906**	**YP_009187477**	**YP_009043593**
62	30139–31557	→	472	Portal protein	**WP_024075266**	**WP_013693867**	WP_023628594		**WP_014717907**		
63	31532–32620	→	362	Head morphogenesis protein	**WP_024075267**	**WP_041931225**	**WP_023628593**		**WP_014717908**	YP_009187475	
64	32634–33104	→	156	Hypothetical protein							
65	33189–33917	→	242	Hypothetical protein	**WP_024075270**	**WP_013693859**	**WP_023628591**		**WP_014717910**		
66	33930–34892	→	320	Major capsid protein					**WP_014717911**	YP_009187473	
67	34918–35529	→	203	Hypothetical protein					**WP_014717912**		
68	35589–36092	→	167	Head-tail connector	WP_024075268	WP_013693861			**WP_014717913**		
69	36110–36481	→	123	Tail attachment protein					**WP_034136295**		
70	36478–37134	→	218	Hypothetical protein	**WP_041161196**	**WP_013693854**	**WP_023628586**		**WP_014717915**	**YP_009187468**	
71	37131–37325	→	64	Hypothetical protein							
72	37325–37741	→	138	Tail terminator protein	**WP_024075276**	**WP_013693853**	WP_023628585		**WP_014717916**	YP_009187466	
73	37814–38470	→	218	Tail protein	**WP_024075278**	**WP_013693851**	**WP_023628584**		**WP_014717917**	**YP_009187465**	
74	38474–38863	→	129	Hypothetical protein	WP_024075279	WP_013693850	WP_023628583		WP_014717918	YP_009187464	
75	38881–39177	→	98	Hypothetical protein	WP_024075280	WP_013693849	WP_031314406		WP_044483156	YP_009187463	
76	39188–42673	→	1161	Tail length tape-measure protein	**WP_024075281**	**WP_013693848**	**WP_023628573**	**WP_025261074**	**WP_014717920**	YP_009187461	YP_009043554
77	42677–43015	→	112	Minor tail protein	**WP_024075282**	WP_013693847	**WP_023628572**	WP_025261073	**WP_014717921**	**YP_009187460**	
78	43027–43779	→	250	Minor tail L	**WP_024075283**	WP_013693846	**WP_023628571**	**WP_025261072**	**WP_014717922**	**YP_009187459**	
79	43851–44018	←	55	Hypothetical protein							
80	44139–44660	→	173	Hypothetical protein							
81	44700–45455	→	251	Tail assembly protein	**AHC35573**	**WP_013693845**	**WP_038706193**	**WP_025261071**	**WP_014717923**	**YP_009187458**	
82	45547–46179	→	210	Hypothetical protein							
83	46238–46660	→	140	Lipoprotein					WP_014718871		
84	46716–47309	→	197	Tail assembly protein I	**WP_024075287**	**WP_013693842**	**WP_038706192**	**WP_025261068**	**WP_014717927**	**YP_009187448**	
85	47347–47554	→	65	Hypothetical protein							
86	47560–47554	→	64	Hypothetical protein							
87	47934–51626	→	1230	Tail fiber protein	**WP_024075288**	**WP_013693841**	**WP_038706191**	**WP_038399956**	**WP_014717928**	**YP_009187445**	**YP_009043558**
88	51626–51952	→	108	Hypothetical protein							YP_009043559
89	52288–52632	→	114	Hypothetical protein							
90	52691–53500	→	269	Tail fiber protein							
91	53497–53889	→	130	Hypothetical protein		WP_013693838			WP_014717932	YP_009187442	YP_009043562
92	53867–54079	→	70	Hypothetical protein							
93	54138–54563	→	141	Peptidoglycan hydrolase		**WP_013693835**			**WP_014717934**		
94	54563–55060	→	165	Rz lysis protein							
95	54708–55022	→	104	Rz1 lysis protein							

^a^*Pseudomonas* sp. TKP (NC_023064, coordinates: 3261951–3321650)

^b^*Pseudomonas brassicacearum* subsp. brassicacearum NFM421 (NC_015379, coordinates: 4653828–4709117); ^c^*Pseudomonas mosselii* SJ10 (NZ_CP009365, coordinates: 2202080–2261265)

^d^*Pseudomonas cichorii* JBC1 (CP007039, coordinates: 41187189–4228131)

^e^*Pseudomonas fluorescens* A506 (CP003041, coordinates: 2195214–2248067)

^f^*Pseudomonas* phage YMC11/02/R656 (NC_028657)

^g^ *Pseudomonas* phage phiPSA1 (NC_024365).

The genome size of the second identified phage, ФAH14b is 16,812 bp and its G+C content (58.9%) is slightly higher than that of the ФAH14a genome. It contained 29 putative genes, of which 10 shared similarity at the amino acid level with other sequences in GenBank (NCBI). Almost all the genes (26) are transcribed rightwards (Fig 3). Putative functional assignments and significant similarities to the predicted genes are listed in Table 2.

**Table 2:** Genes located within the ФAH14b genome and proteins homologous to the ФAH14b-encoded proteins found in selected *Pseudomonas* spp. genomes and Enterobacteria phage P4. Searches were performed with the following cut offs *E* value < 1e-10 and < 1e-40. The hits obtained with e-value 1e^-40^ are bolded.

ORF no.	Coding region (bp)	Strand	Protein size (aa)	Possible function	*Pseudomonas* sp. TKP^a^	*Pseudomonas mosselii* SJ10^b^	*Pseudomonas cichorii* JBC1^c^	*Pseudomonas fluorescens* NCIMB 11764^d^	*Pseudomonas azotoformans* S4^e^	Enterobacteria phage P4^f^
1	233–1438	→	401	Integrase XerC	**WP_024072879**	**WP_023629283**	**WP_025258583**	**WP_017336050**	**AMN78275**	**NP_042035**
2	1597–2553	→	318	HflK/C family protein						
3	2679–2939	→	86	AlpA regulatory protein	WP_051448912	WP_019750501	WP_025258581	WP_031318270		**NP_042041**
4	2940–3305	→	121	Hypothetical protein				WP_017336047		
5	3302–3799	→	165	Phage regulatory protein Rha	**WP_024072875**		**AHF66019**	**WP_031318268**		
6	3771–4253	→	160	Hypothetical protein	AHC32948			**WP_033037162**		
7	4250–4465	→	71	Hypothetical protein						
8	4462–4752	→	96	Hypothetical protein	WP_024072873					
9	4749–4985	→	78	Hypothetical protein						
10	4982–5485	→	78	Hypothetical protein						
11	5482–5607	→	167	Hypothetical protein						
12	5618–5902	→	94	Hypothetical protein	**WP_024072871**		**WP_025258573**	**WP_017336043**	**AMN78270**	
13	5905–6360	→	151	Hypothetical protein						
14	6353–6580	→	75	Hypothetical protein	**WP_024072870**				**AMN78269**	
15	6577–7458	→	293	Topoisomerase-primase	**WP_024072869**	**WP_052062225**	**WP_025258572**	**WP_017336041**	**AMN78268**	**NP_042036**
16	7445–9247	→	600	RNA helicase	**WP_024072868**		**WP_025258571**	**WP_017336040**	**AMN78267**	
17	9557–9892	→	111	Hypothetical protein	**AHC32941**			**WP_031318265**	**AMN78266**	
18	9889–10497	→	202	Hypothetical protein	**WP_024072866**					
19	10604–10984	→	126	Hypothetical protein	**WP_024072865**			**WP_017336038**	**AMN78265**	
20	10997–11599	→	200	Tail assembly protein I	**WP_024072864**	**WP_023629288**		WP_017336037	**AMN78264**	
21	11639–12061	→	140	Lipoprotein	**AHC32937**			WP_017336036	**AMN78263**	
22	12470–13594	→	374	Histidine kinase						
23	13706–13822	←	38	Hypothetical protein						
24	13987–14187	→	66	Transcriptional regulator						
25	14703–14885	←	60	Hypothetical protein						
26	15110–15367	→	110	Hypothetical protein	**WP_024072860**			**WP_031318262**		
27	15899–16030	←	43	Hypothetical protein						
28	16114–16314	→	66	Hypothetical protein	WP_024072858			WP_031318262		
29	16390–16587	→	65	Hypothetical protein						

^a^*Pseudomonas* sp. TKP (NC_023064; coordinates: 417067–434373)

^b^*Pseudomonas mosselii* SJ10 (NZ_CP009365; coordinates: 3922068–3938323)

^c^*Pseudomonas cichorii* JBC1 (CP007039; coordinates: 948505–967433)

^d^*Pseudomonas fluorescens* NCIMB 11764 (CP010945; coordinates: 1504120–1518971)

^e^*Pseudomonas azotoformans* S4 (CP014546; coordinates: 1711898–1724788)

^f^Enterobacteria phage P4 (NC_001609).

An interesting observation is the presence of two imperfect inverted repeats in the ФAH14a genome. These regions (coordinates 13999–14480 and 46815–47308, respectively) show 76% of reciprocal identity and comprise a continuous section of 50 identical nucleotides. Genes *AH14a_p32* and *AH14a_p84* are located within these regions. The predicted proteins encoded by the *AH14a_p32* and *AH14a_p84* genes, annotated as tail assembly protein I, share 79% of amino acid identity. Interestingly, the aforementioned two segments of the ФAH14a genome also share similarity with a part of the ФAH14b genome (coordinates 11054–11599), which also encodes a putative tail assembly protein I (AH14b_p14; 71% and 74% identity with AH14a_p32 and AH14a_p84, respectively). Pfam and HHPred analyses confirmed that the predicted proteins belong to the group of homologs of the bacteriophage λ tail assembly protein I. This family consists of TAPI proteins from lambdoid T1 phages and related prophages, and their members contain a core ubiquitin fold domain. The exact function of TAPI is yet unknown, however it was shown that it is not incorporated into the mature tail, but is rather processed by a specific peptidase [36].

On the other hand, the lack of any other resemblance between ФAH14a and ФAH14b strongly suggests that the smaller phage is not a result of genetic degradation of the larger ФAH14a.

### Functional assignments for the predicted ФAH14a-encoded proteins

The ФAH14a genome contained several genetic modules, including those responsible for integration, DNA methylation, DNA recombination, transcription regulation, replication, DNA packaging, capsid morphogenesis and lysis of the host cell. The order of these genes and gene clusters is similar to the order of the cognate modules in other tailed phages.

The *AH14a_p01* gene is predicted to encode an integrase, as its protein product belongs to the tyrosine recombinase family (pfamPF00589). The most closely related virus-encoded protein, Gp1 (GenBank accession YP_004306367) of P2-like phage KS5 of *Burkholderia cepacia* [37] shares 32.9% identity with the AH14a_p01 protein. No genes coding for excisionases have been identified within the ΦAH14a genome.

Based on the BLASTp similarity analysis, the AH14a_p05 protein has been assigned to the DNA methyltransferase family. Its potential enzymatic activity has been experimentally tested (see below).

Temperate phages possess genes responsible for switching between the lytic and the lysogenic cycles [38, 39]. Such a control region, composed of genes homologous to the *cI* and *cro* repressor genes of the phage λ, transcribed in opposite directions, was found in ФAH14a. The predicted prophage CI repressor is encoded by *AH14a_p39*. The protein shows 60.2% identity with the repressor PrtR of the *Pseudomonas* phage DVM-2008 (ACH86126). It belongs to the XRE family of transcriptional regulators (COG2932) and its amino-terminal region consists of a helix-turn-helix domain (pfam01381), while its carboxyl-terminal region contains the S24 signal peptidase domain (pfam00717). An inversely oriented Cro-like protein that represses genes normally expressed in the early stage of phage development and which is necessary for the late stage of lytic growth, is encoded by *AH14a_p40*. Its homologs are encoded by other *Pseudomonas* phages, e.g., PMG1, D3 [40] and phi297.

The DNA replication machinery of ΦAH14a presumably comprises AH14a_p44 (containing ParBC nuclease domain, pfam02195) and AH14a_p45 (containing a helix-turn-helix motif, pfam13730). The AH14a_p45 protein exhibits a significant sequence identity with several proteins described in the NCBI database as phage replication proteins. Among the AH14a_p45 homologs, there are also replication proteins of five functional viruses, i.e. *Pseudomonas* phages H66 and F116 [41], *Flavobacterium* phage FCL-2 [42], *Psychrobacter* phage pOW20-A and *Mannheimia* phage vB_MhS_587AP2 [34].

Terminase enzymes are essential for packing of the phage genome DNA into the phage head and typically comprise small and large subunits (TerS and TerL, respectively). TerS has DNA-binding activity, and TerL provides ATP-binding and DNA cleavage activities [43]. A putative TerL (AH14a_p61), shared similarities with TerL of a *Rhizobium* phage RHEph10 (35% identity) [44]. We were not able to identify the small terminase subunit in the ФAH14a genome. The most probable candidate for that role is AH14a_p60, which has the appropriate size and genomic location (the *terS* gene is typically located upstream, and is transcribed in the same direction as *terL*).

The gene cluster encoding phage structural proteins is typically located adjacent to the DNA packaging module, and usually begins with the portal and head morphogenesis genes, followed by the tail morphogenesis genes. The predicted structural gene cluster of ФAH14a covers the ORFs from *AH14a_p62* (predicted as a putative portal protein) to *AH14a_p90*, and lies adjacent to the host cell lysis module. We were able to assign the predicted structural function to 14 of 29 proteins encoded within this module, including: portal protein (AH14a_p62), head morphogenesis (AH14a_p63), major capsid (AH14a_p66), head-tail connector (AH14a_p68), tail attachment (AH14_69), tail terminator (AH14_72), tail (AH14a_p73, _p77, _p78), tail length tape-measure (AH14_76), tail assembly (AH14a_p81 and p_84), and tail fiber (AH14a_p87 and p90). All these putative proteins shared sequence similarity with the structural proteins identified in phages of *Salmonella*, Enterobacteria, *Psychrobacter*, *Pseudomonas* and others. One of the largest putative structural protein products of ФAH14a (1161 amino acid residues) is encoded by *AH15a_p76* and is 38% identical with putative tape measure proteins (TMPs) of *Pseudomonas* MP48, PA1phi, JBD5, H70, JD024, LPB1 and ФPSA1 phages, all belonging to the *Siphoviridae* family. TMPs are responsible for precise determination of the tail shaft length and is present in all the long-tailed phages [45].

**Fig 4:**
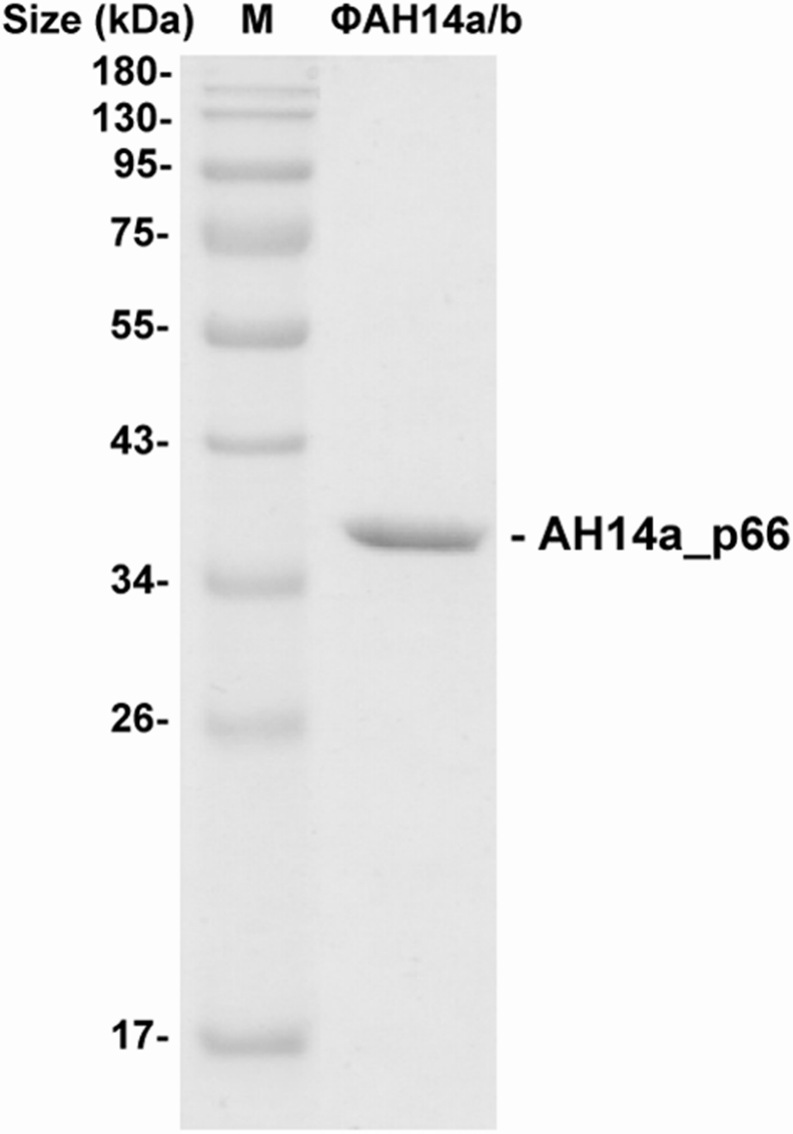
Separation of ΦAH14a and ΦAH14b virion proteins by SDS-polyacrylamide gel electrophoresis (12%). M–Page Ruler prestained protein ladder SM0671 (Thermo Scientific).

To confirm which ФAH14a ORFs encode components of its viral coat, CsCl-purified phage particles were resolved by SDS-PAGE. This revealed only one protein band, which was then examined by mass spectrometry (Fig 4). LC-MS/MS analysis identified a putative major capsid protein (AH14a_p66) with the sequence coverage of 92%. For the identification of the other possible virion proteins, a SDS-PAGE gel was systematically sliced, and the proteins present within each slice were subjected to MS identification. Three additional proteins were detected in this way: AH14a_p62 (putative portal protein), _p63 (putative head morphogenesis protein) and _p68 (putative head-tail connector) with the sequence coverage of 12%, 17% and 10%, respectively. The presence of only the head structural proteins in the proteome analysis of the ФAH14a virion was not surprising. On electron micrographs, only heads lacking any tail structures could be detected (Fig 1). Based on sequence comparisons with the completely annotated phage genomes, ФAH14a most probably carries information necessary for tail and fibers formation (Table 1 and see above), but for unknown reasons the tail assembly or its attachment to the capsid head is impaired. The incomplete assembly of viral particles could be the simplest explanation of the observed inability of any phage to lytic growth on the tested *Pseudomonas* spp. hosts (see above). Nevertheless, the presence of only one dominant protein band in the proteome analysis of the viral particles isolated from *Pseudomonas* sp. ANT_H14 is a strong evidence that both ФAH14a and ФAH14b genomes are encapsidated in the virion particles built of AH14a_p66 capsomers.

The most intriguing gene in the structural gene cluster is *AH14a_p83* which putative protein product contains an N-terminal sorting signal that favors translocation into the outer membrane conforming to the Prosite consensus for prokaryotic lipoprotein lipid attachment sites (amino acids 1–16: MRILIAAVAVAMLAGC; potential lipidation site, C16 is underlined) [46, 47]. In prokaryotes, membrane lipoproteins are synthesized with a precursor signal peptide, which is cleaved by a specific lipoprotein signal peptidase (signal peptidase II). The peptidase recognizes a conserved sequence and cuts upstream of a cysteine residue, to which a glyceride-fatty acid lipid is attached [46]. Lipoproteins (encoded by the *cor* genes) are found in a number of phages, in which they prevent superinfection by inactivating the receptors. For example, a protein product of the N15 gene *24*, the homolog and functional analog of the Cor phage ϕ80 [48] is responsible for the inability of N15 lysogens to adsorb FhuA-dependent bacteriophages N15, T1, and ϕ80 [49–51]. The *cor* genes of N15, ϕ80 and Rtp phages are located next to the tail fiber genes [49]. Similarly, in the phage ФAH14a, *AH14a_p87* encoding a putative tail fiber is located in close proximity to a lipoprotein gene *AH14a_p83*.

Non-filamentous bacteriophages release their progeny by lysing the host cell. We suppose that ΦAH14a may use the protein product of *AH14a_p93* for such a purpose. AH14a_p93 shares similarity with other *Pseudomonas* phage lytic enzymes, e.g. vB_PaeP_Tr60_Ab31 [52]. Its potential enzymatic activity has been experimentally tested (see below). We have also identified putative equivalents of two accessory lysis genes Rz/Rz1, encoded by the *AH14a_p94* and *AH14a_p95* genes, respectively. Similarly to the λ Rz and Rz1 lysis genes, Rz1 is completely embedded in the +1 register within Rz [53]. None of the ФAH14a ORFs share homology with holins, which play a role in the timing of cell lysis by inducing non-specific lesions in the cytoplasmic membrane. [54, 55].

### Functional characterization of DNA methyltransferase of ΦAH14a prophage

The AH14a_p05 protein showed a high similarity to a large number of uncharacterized proteins annotated as putative DNA methyltransferases (MTases) with the predicted sequence specificity CCCGGG, including M.PliPIgORF20415P of *Pseudomonas libanensis* (KPG72873, 87% identity). Among its homologs there is also the plasmid-encoded *C*^*4*^*-*methyl-cytosine (m^4^C) M.Pac25I (AAD40332, 44% identity) of *Pseudomonas alcaligenes* NCIB 9867, whose target motif (CCCGGG) was experimentally identified [56].

**Fig 5:**
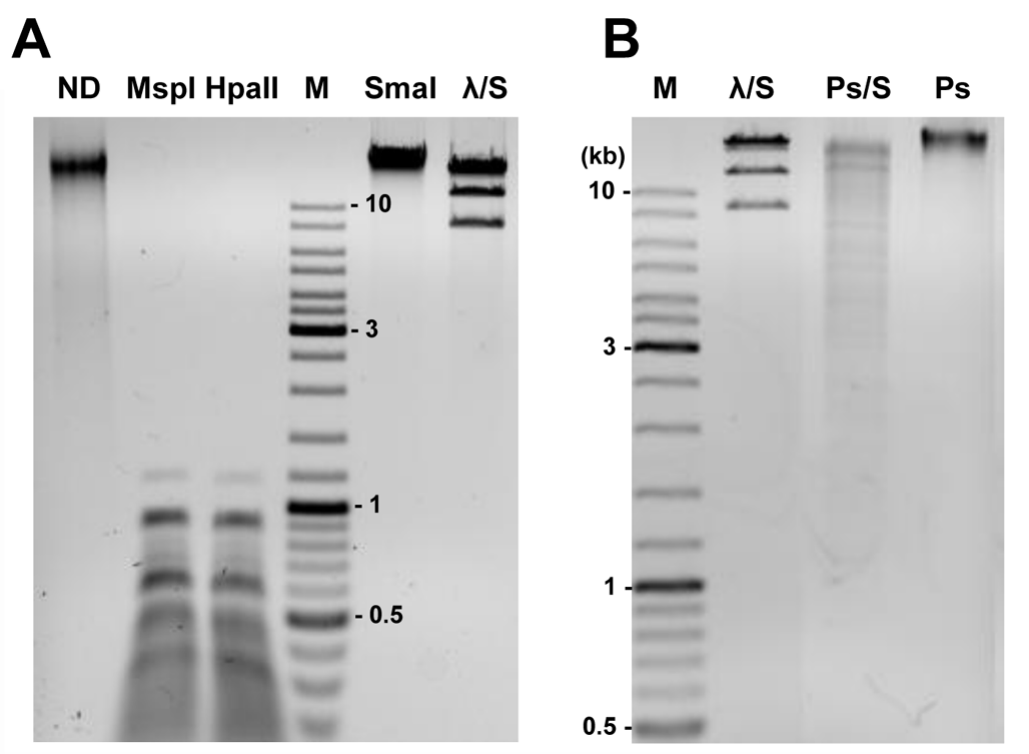
**Restriction patterns of DNAs**: isolated from viral particles (a mixture of ФAH14a and ФAH14b genomic DNA) cleaved with selected REases: HpaII (CCGG), MspI (CCGG) and SmaI (CCCGGG) [panel A] and *Pseudomonas* sp. ANT_H14 genomic DNA cleaved with SmaI (CCCGGG) [panel B]. Digest mixtures were electrophoresed on 0.8% agarose gels and stained with ethidium bromide. ND–undigested DNA isolated from viral particles; λ/S–DNA of λ *dam*^*-*^ *dcm*^*-*^ digested with SmaI; Ps–undigested *Pseudomonas* sp. ANT_H14 genomic DNA; Ps/S–ANT_H14 genomic DNA digested with SmaI; M–GeneRuler 100–10,000 bp size marker.

The specificity of AH14a_p05 was tested by comparative digestion of the pET-AH14a_p05 plasmid DNA, isolated from IPTG-induced and uninduced *E*. *coli* cultures, with SmaI (CCCGGG), BsuRI (GGCC), HpaII (CCGG), Bsh1236I (CGCG) and Hin6I (GCGC) restriction enzymes. The DNA of pET-AH14a_p05 isolated from the induced culture was cleaved by all the tested restriction enzymes, with the exception of SmaI. In contrast, the pET-AH14a_p05 DNA isolated from the non-induced culture was susceptible to all restriction enzymes, including SmaI. The DNAs of ΦAH14a, with sixteen CCCGGG sites, and ΦAH14b, with seven sites, were completely resistant to SmaI digestion, but were sensitive to HpaII and MspI REases (Fig 5A). According to REBASE [27], SmaI is sensitive to m^4^C methylation of any cytosine in its recognition sequence (CCCGGG). HpaII and MspI both recognize the CCGG sequence and when the outer C in their cognate sequence is methylated to m^4^C, they cannot cleave. In addition, HpaII is unable to cut DNA when the inner cytosine is methylated to m^4^C [57]. As both HpaII and MspI cut their targets in these phage DNAs it is clear that neither second nor third cytosine in the sequence CCCGGG is methylated by AH14a_p05. Instead, we concluded that AH14a_p05 modifies the first cytosine in its target sequence.

In bacteria, the major role of DNA methylation is to protect their DNA against degradation by restriction enzymes [58]. However, it does not seem that ФAH14a uses AH14a_p05 to overcome this type of host protection, as the *Pseudomonas* sp. ANT_H14 genomic DNA turned out to be susceptible to cleavage by SmaI REase (Fig 5B). This excludes the possibility that this strain carriers an active restriction-modification system with the CCCGGG specificity. Some prokaryotic DNA MTases participate in the regulatory events of DNA replication or transposition [59]. Interestingly, the adjacent gene AH14a_p06 encodes a putative transcription regulator and together with ΦAH14a DNA MTase and integrase genes they are located in a cluster of genes transcribed leftwards on the genetic map. Nevertheless, the involvement of this enzyme in the regulation of viral or bacterial genes has to be further investigated.

### Functional characterization of the putative lytic enzyme of the ΦAH14a prophage

**Fig 6:**
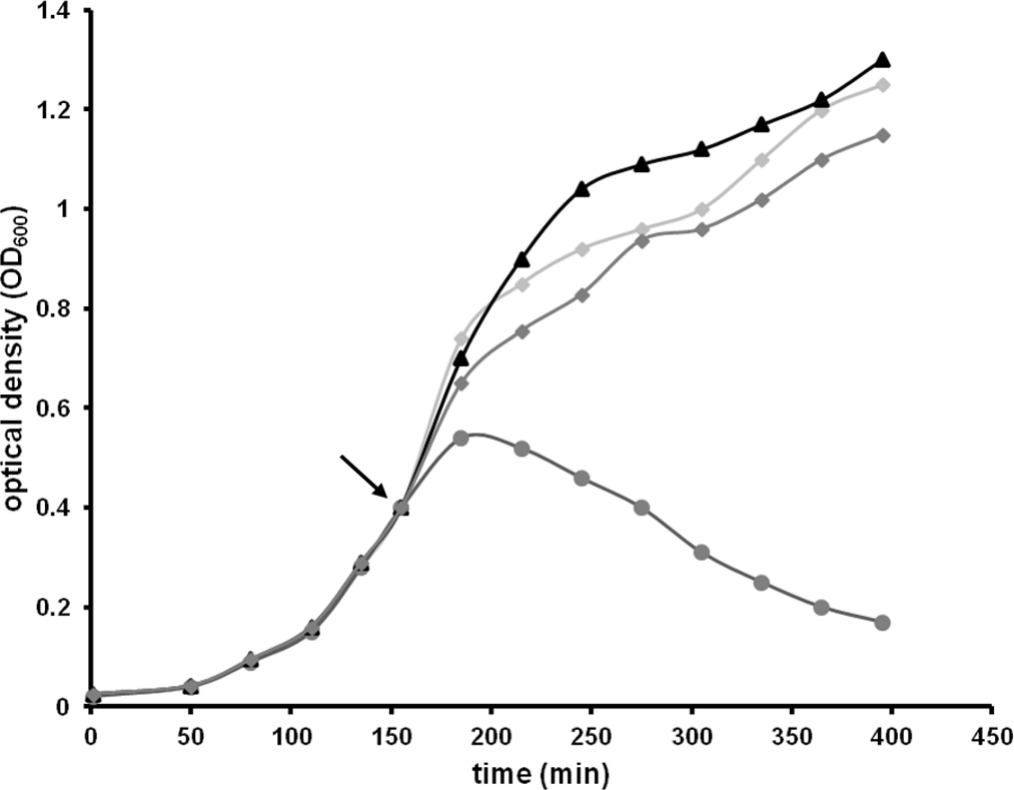
Profiles of *E*. *coli* cell lysis as the result of AH14a_p93 expression. A ER2566(pET-AH14a_p93) culture was grown at 37°C to exponential phase (OD_600_ of 0.4). Untreated cultures (with glucose, but without IPTG, indicated by triangles) or cultures induced by the addition of IPTG to a final concentration of 1 mM (circles) were monitored for growth. A ER2566(pET-AH14a_p05) culture was used as the control in a parallel test (not induced—squares, induced—diamonds). The arrow indicates the time at which IPTG was added. OD–optical density.

To confirm that the *AH14a_p93* gene indeed encodes a functional peptidoglycan hydrolase enzyme, we cloned the *AH14a_p93* gene in *E*. *coli* under the control of an inducible T7 promotor. As shown in Fig 6, the induction of the putative hydrolase gene by IPTG had a lethal effect on the heterological host, resulting in cell lysis. It should be stressed that the activity of AH14a_p93 (or any other ΦAH14a lytic protein) was not manifested in the native strain, as we did not observe cell lysis following mitomycin C treatment.

### Functional assignments for the predicted ФAH14b-encoded proteins

Based on the *in silico* analysis, we were able to assign putative biological functions to 10 of the ФAH14b ORFs. The AH14b_p01 protein shows homology to the tyrosine recombinase encoded by the *Stenotrophomonas* phage S1 (46.8% identity) [60], the *Pseudomonas* phage F116 (42.4% identity) [41] and the Enterobacteria phage P4 (40.2% identity) [61, 62]. Therefore, we suggest that it is an integrase.

The AH14b_p02 protein belongs to SPFH (stomatin, prohibitin, flotillin, and HflK/C) superfamily (cl19107), in which prokaryotic members HflK/C were shown to play a role in the switching between lysogenic and lytic cycle growth during phage infection. The *E*. *coli* membrane proteins HflK and HflC form a complex HflKC that was found to act as a modulator of the HflB(FtsH)-mediated proteolysis of λ CII, which is the key element regulating the switch between lytic and lysogenic lifecycle through the activation of several phage λ promoters [63].

The AH14b_p03 protein is classified as a member of the AlpA family transcriptional regulators (pfam05930). The AlpA of *E*.*coli* defective prophage CP4–57 (the abbreviation stands for ‘cryptic P4-like prophage at min 57’) is a key transcriptional regulator (activator) for the integrase IntA [64]. It was also shown that during the natural development of *E*. *coli* biofilms, the AlpA expression is induced up to 11-fold [65], and this induction leads to CP4–57 excision, which in turn is beneficial to the process of biofilm formation [66]. The AlpA-like AH14b_p03 protein bears 21% identity to prophage CP4-57 AlpA (WP_009604557) and 24% to ORF88 of the phage P4 (NP_042041). The distance between the putative integrase gene of ΦAH14b (*AH14b_p01*) and the AlpA-like *AH14b_p03* is also similar to that between the *intA* and *alpA* genes in the prophage CP4-57 (approximately 1,200 and 1,100 bp, respectively).

Pfam search demonstrated that AH14b_p04 belongs to the phage regulatory protein Rha family (PF09669). Among its members, there are the Rha antirepressor of the phage ϕ80 and the product of the late operon *rha* (*orf201*) gene of the phage P22, which is detrimental for lytic growth in the absence of the integration host factor (IHF) function, which regulates the *rha* gene [67–69]. In other words, the Rha protein blocks phage growth during infections of IHF defective hosts.

The AH14b_p15 protein contains the topoisomerase-primase nucleotidyl transferase/hydrolase domain (pfam PF13362) found in the active site regions of bacterial DnaG-type primases and their homologs. Primases synthesize RNA primers for the initiation of DNA replication. DnaG type primases are often closely associated with DNA helicases in primosome assemblies. AH14b_p15 is in 42% identical with the putative P4-specific DNA primase (NP_042036), but is much shorter (293 aa *versus* 777 aa). Perhaps, AH14b_p15 works in association with AH14b_p16, which shares sequence similarity with bacterial helicases.

As mentioned above, AH14b_p20 is homologous to AH14a_p32 and AH14a_p81, both of which were assigned as tail assembly proteins I. Similarly, AH14b_p21 is homologous to AH14a_p82 (42% identity), assigned as a membrane lipoprotein (see above), whose putative protein product contains an N-terminal sorting signal that favors translocation into the outer membrane (MRRIATTALFAALLAGC, amino acids 1–17; potential lipidation site, C17 is underlined).

High sequence similarity (up to 84.5% identity) was found between the AH14b_p22 protein and bacterial histidine sensory kinases. Proteins from this group belong to bacterial two-component regulatory systems, which transmit environmental signals into the bacterial cell, in order to modulate gene activity [70]. Finally, the *AH14_p24* gene encodes a protein showing 41.4% amino acid identity to gp16 of the *Burkholderia* phage BcepMu, for which DNA-binding activity is suggested [71].

The other remaining nineteen ΦAH14b genes encode proteins showing similarity exclusively to uncharacterized homologs. None of these putative proteins were detected in the capsid, which suggests that they are not involved in structural functions. The results of mass spectrometry analysis (see above) demonstrated that all the identified virion proteins are encoded by the ΦAH14a genes. This strongly indicates that the ΦAH14b genome was encapsidated in the same virion particles as ΦAH14a.

We also were unable to identify any genes in the ΦAH14b genome, whose protein products could function as terminases or lytic enzymes. However, ΦAH14b has its own DNA primase, hence it is probably capable of autonomous replication. All the other proteins necessary for the ΦAH14b virion formation, DNA packaging, and host cell lysis have to be provided *in trans* by a helper phage.

### Sequence similarity between ФAH14a and ФAH14b and other *Pseudomonas* phages

The ФAH14a genome shares limited nucleotide sequence identity with only two genomes of viruses currently available in the NCBI viral database, i.e. the temperate phage of *Pseudomonas syringae* pv. actinidiae ФPSA1 [(GenBank NC_024365), 74% identity within 602 bp region, coordinates 9138–9740] [72] and an uncharacterized *Pseudomonas* phage YMC11/02/R656 [(GenBank KT968831), 77% identity within 2312 bp region, coordinates 27793–30110]. BLASTP analyses showed that the ФAH14a proteome had 12 homologs with ФPSA1 and 22 with YMC11/02/R656 (Table 1).

The ФAH14b virus can be considered unique as its comparison with the phage genomic sequences available in the NCBI viral database showed no discernible DNA sequence similarity to any of them.

In the course of this study several putative prophage sequences related to ФAH14a and ФAH14b were detected in the genomes of *Pseudomonas* sp. TKP, *Pseudomonas brassicacearum* subsp. brassicacearum NFM421, *Pseudomonas cichorii* JBC1, *Pseudomonas mosselii* SJ10, *Pseudomonas azotoformans* S4, *Pseudomonas fluorescens* NCIMB 11764 and A506 (Tables 1 and 2). For instance, comparative analysis revealed that ФAH14a had 33 similar proteins with a putative prophage of *P*. *brassicacearum* subsp. brassicacearum NFM421 (NC_015379, coordinates; 4653828–4709117), 27 with *Pseudomonas* sp. TKP (NC_023064, coordinates: 3261951–3321650) and the homologies were mainly located in a collinear cluster of genes on the right arm of the ФAH14a genome and included a terminase, as well as portal, structure and morphogenesis proteins (except the major capsid protein). Amino acid sequence similarity of the same functional modules was also identified between ФAH14a and the previously mentioned *Pseudomonas* phage YMC11/02/R656.

It is worth mentioning that within some of the aforementioned genomes (*Pseudomonas* sp. TKP, *P*. *cichorii* JBC1 and *P*. *mosselii* SJ10) the ‘accompanying’ prophage sequences, similar to ФAH14b, were also found (Table 2). Interestingly, the ФAH14b-like region in *Pseudomonas* sp. TKP genome is flanked by duplicated 18 bp sequences (5’-GTTCGATTCCGTCTCTGG-3’), which most probably constitute attachment sites of the presumed prophage. An identical sequence (found in the same localization upstream the integrase gene) is present in ФAH14b (coordinates: 1–18).

Fourteen homologous proteins are also shared between ФAH14b and a putative prophage found in *P*. *fluorescens* NCIMB 11764 (Table 2), but we were unable to detect a counterpart of ФAH14a in its genome.

### Classification of the ANT_H14 prophages according to their neck organization

Tail morphology serves as a basis to classify *Caudovirales* phages into three distinct families: *Myoviridae* bearing complex contractile tails, *Siphoviridae* with long, noncontractile tails, and *Podoviridae* with short tails. As we could not use TEM analysis for a direct classification of the ANT_H14 prophages, VIRFAM analysis was used instead [32]. VIRFAM is a webserver that automatically identifies proteins of the phage head-neck-tail module and assigns phages to the most closely related cluster of phages collected in the Aclame database [32, 73].

VIRFAM predicted ФAH14a to be a member of the *Siphoviridae* type 1 group, clustered with φHSIC of *Listonella pelagia*, *Salmonella* phages: KS7 and SETP3, *Pseudomonas* phages: M6 and PA73, and *Burkholderia* phage BcepGomr (Cluster 5). Cluster 5 of the Neck Type 1 adopts the structural organization of the *Siphoviridae* phage SPP1 neck (PA73-like). The gene order in the genomes of the γ-Proteobacteria phages of Cluster 5 is as follows: terminase, portal, MCP, head-completion, head-closure and tail-completion genes (in ФAH14a: *AH14_p61*, *_p62*, *_p66*, *_p68*, *_p69*, *_p72*, respectively).

The identification of all the potential components of the capsid neck (interface between head and tail) and, substantially, all the elements required for tail assembly (Table 1) suggests that the observed incomplete virus particles (tail-less) probably do not result from the loss of the coding sequence(s). Their appearance is rather caused by mutation(s) in a structure/morphogenesis gene or in a regulatory element located downstream of the *AH14_p66* gene (encoding a major capsid protein), which cannot be detected by bioinformatic analysis.

The VIRFAM analysis did not identify any head-neck-tail proteins in the ФAH14b proteome therefore this prophage could not be assigned to any of the 4 canonical types characterized by Lopes et al. [32]. This analysis supports the earlier conclusion that this phage lacks genes encoding structural proteins.

### ΦAH14a-ΦAH14b as a novel helper-satellite system

Based on our observations, we suggest that ΦAH14b might be a satellite-like phage, which is defined as a virus that has a life cycle dependent on a helper virus, in this case on ΦAH14a. Satellite viruses lack extensive nucleotide sequence homology to the helper virus and are dispensable for helper virus proliferation [74]. The best studied satellite phage is the temperate coliphage P4, which lacks all of the genetic information necessary for capsid, tail and lysis functions, and is therefore dependent on a helper phage, such as P2, for lytic propagation [75].

No significant homology was found between the ΦAH14a and P2 phage proteins. However, three ΦAH14b proteins, i.e. AH14b_01 (integrase), AH14b_03 (AlpA-like transcription regulator) and AH14b_14 (primase), share similarity with respective proteins encoded by P4. We did not detect any ORFs homologous to the other experimentally tested P4 genes, e.g. *cII*, *gop*, *psu*, and *sid*, whose protein products are involved in capsid size determination (see below).

Both P4 and its helper phage P2 capsids are made of the same major capsid protein encoded by P2. P4 is able to control the subunit assembly into virions [76, 77]. The protein product of the P4 *sid* gene causes the P2 capsid proteins to assemble into smaller heads that are about 1/3 the size of those normally synthesized by P2 itself, corresponding to the difference in the size of the genomes (11.6 kb *versus* 33.5 kb). However, P4 mutants with a defective *sid* gene despite being unable to form small capsids, remain viable. They package two or three copies of P4 DNA into P2-size capsids [77, 78]. P2 mutants with the same phenotypic effect have also been isolated [79].

In another known satellite/helper system, genetic elements SaPIs of *S*. *aureus* are mobilized by specific helper phages e.g. 80α [8, 80] and are packaged into phage-like transducing particles, which are often smaller than the native helper virions [7, 8]. For instance, SaPI1 (using protein products of its *cmpA* and *cmpB* morphogenetic genes) redirects the phage capsid assembly pathway [81]. On the other hand, SaPI1 *cpmA*-*cmpB* mutants and some of the naturally occurring SaPIs derivatives, such as SaPIbov5, which lack the SaPI packaging module, do not produce small capsids [7]. These SaPIs are packaged into the full-sized phage capsids. It was predicted that a concatemer containing three tandem copies of the standard 15-kb SaPI genome is carried by the helper-sized particles [82].

Our results indicate that the ΦAH14b genome is packed into viral particles made of proteins encoded by ΦAH14a. Morphological studies using TEM showed that not only all viral particles had the same morphological features, i.e. hexagonal tail-less heads, but also had the same size, which strongly suggests that both ΦAH14a and ΦAH14b genomes were covered with the same protein coats.

As ΦAH14b lacks information for structural proteins, we hypothesize that ΦAH14b parasitizes on ΦAH14a utilizing its capsids. However, it does not seem that ΦAH14b is capable of altering ΦAH14a capsid head morphology, therefore its phenotype is analogous to P4 mutants with defective *sid* genes (see above).

Surprisingly, these viral particles appear to be incomplete as they are devoid of tails and fibers, even though ΦAH14a contains the coding information for these structures. It seems that, for an unknown reason, ΦAH14a is unable to carry out a complete virion assembly. It cannot be ruled out that ΦAH14a is already undergoing degenerative changes towards a defective prophage. Recent data suggests that prophage sequences are subject to accumulation of inactivating mutations, followed by genetic degradation. However, at the same time, phage-specific adaptive functions that are advantageous for the host (i.e. immunity to superinfection) are conserved [4]. ΦAH14a might constitute an interesting case for studies of such regressive evolution, and therefore might be helpful in understanding the evolution of phages as parasites and their ‘domestication’ by bacterial hosts.

On the other hand, the putative satellite phage ΦAH14b retained the ability to simultaneous induction with the helper and to package its DNA within the ΦAH14a protein coat. The maintenance of the native functions by a satellite virus, while its supporting helper phage is unable to produce fully-active virions seems futile. This apparent paradox can be explained in two ways: (i) defectiveness of ΦAH14a is the result of a very recent evolutionary event or (ii) mutations in the ΦAH14b prophage sequence undergo strong purifying selection and are not stored in the *Pseudomonas* sp. ANT_H14 population. The latter allows for another suggestion that ΦAH14b might somehow provide advantageous phenotype to the host (in contrast to ΦAH14a), e.g. by encoding features, which make a significant contribution to the host fitness. Although this is only a speculation we hypothesize that the presence of a histidine kinase (AH14b_p22) and AlpA (AH14b_p03) might be potentially beneficial for the host. As mentioned above, the AlpA homolog of AH14b_p03 encoded by the CP4-57 prophage is involved in *E*.*coli* biofilm formation [66]. A similar phenomenon of dependency of the biofilm life cycle was also shown for *Pseudomonas aeruginosa*. In this case not only the phenotypic variation of the bacterial biofilm, but also virulence is dependent on a filamentous prophage, Pf4 [83, 84].

## Conclusions

In this work two novel prophages, ФAH14a and ФAH14b, of a cold-active *Pseudomonas* sp. ANT_H14 have been identified and their genomes have been described. Both, ФAH14a and ФAH14b are packed into the same tail- and fiber-less capsids, build of the ФAH14a-encoded major capsid protein. They constitute a putative helper-satellite system, in which ФAH14b seems to parasitize ФAH14a. Although the phenomenon of molecular piracy seems to be common in viruses, P2/P4 and SaPI/80α remain the only two examples, which have been extensively studied. Therefore, this is the first report on the possible existence of a helper-satellite system in pseudomonads. Moreover bioinformatics analysis indicates that the ΦAH14a-ΦAH14b duo is probably not a unique set in this genus, as we were able to identify homologous pairs in other *Pseudomonas* strains.

It can be concluded that the performed characterization of the ΦAH14a and ΦAH14b duo may provide a starting point for further exploration of similar *Pseudomonas* systems and for advanced comparative analyzes aimed at restoring the full functionality of the ΦAH14a prophage. Rebuilding of ΦAH14a infectivity could give us a hint how its degenerative changes have proceeded over time.
